# Posttraumatic Stress Disorder Among Residents of Conflict-Affected Towns

**DOI:** 10.1001/jamanetworkopen.2023.29156

**Published:** 2023-08-16

**Authors:** Solomon Moges, Gebeyaw Molla, Kindie Mekuria, Niguss Molla, Fentaw Girmaw

**Affiliations:** 1 College of Health Sciences, Woldia University, Woldia, Ethiopia; 2 Faculty of Education and Behavioral Sciences, Woldia University, Woldia, Ethiopia; 3 Department of Pharmacy, College of Health Sciences, Woldia University, Woldia, Ethiopia

## Abstract

This cross-sectional study assesses the prevalence of and risk factors associated with posttraumatic stress disorder in a large conflict-affected area in Ethiopia.

## Introduction

In the last several decades, many populations have been affected by disasters such as war.^1^ Posttraumatic stress disorder (PTSD) is one of the mental health problems associated with war.^2^ According to the *Diagnostic and Statistical Manual of Mental Disorders* (Fifth Edition) (*DSM-5*), PTSD occurs after exposure to a traumatic or stressful event and is characterized by symptoms of intrusion, avoidance, mood, cognition alternation, and hyperarousal lasting more than a month.^3^ Situations such as increased exposure to traumatic experiences, repeated and continuous exposure to violence, armed conflict, and mass-casualty events, together with the absence of access to mental health treatment, significantly affect the burden of PTSD.^4,5^ This study covers large conflict-affected areas. Therefore, we sought to assess the prevalence and risk factors associated with PTSD.

## Methods

The institutional review board of Woldia University approved this community-based cross-sectional study. Before the interview, verbal informed consent was obtained from the study participants; the study adhered to the STROBE reporting guideline. Adults aged 18 and older residing in the selected conflict-affected towns of North Wollo and Wag Himra Zones were included. Data were collected from May to June 2022. Individuals who were seriously ill, mentally incompetent, or had a physical disability were excluded. We determined PTSD using the Posttraumatic Stress Disorder Checklist for *DSM-5* (PCL-5). We used logistic regression analyses to assess PTSD and its risk factors. Data were analyzed using SPSS, version 25 (IBM). We considered statistical significance at a *P* < .05.

## Results

This study included 601 participants, 304 were male and 297 (49.4%) were female, with a response rate of 98.5%. Among these, 239 respondents (39.8%) were aged 25 to 34 years and 144 (24%) were aged 45 years or older. Regarding religion, 505 (84.0%) were Orthodox Christians, and 94 (15.6%) were Muslim. Of the participants, 180 (30.0%) were government-employed workers. Nearly one-fifth of the study participants (104 [17.3%]) were students, jobless, and daily laborers, and 330 participants (54.9%) had a monthly income of less than 2995.35 Ethiopian birr (US $52.27).

More than one-third of the participants (263 [43.8%]), had been displaced more than once due to the conflict. Nearly three-fourths of the participants (442 [73.5%]) witnessed the murder of a family member or friend (Table 1). Being female was associated with decreased risk of PTSD when compared with being male. Of the participants, 146 (24.3%) were reported having PTSD (95% CI, 21%-27.8%). In the current study, we found that variables such as being female (AOR, 0.48; 95% CI, 0.27-0.86), experiencing torture (AOR, 1.70; 95% CI, 1.02-3.08), being in a war-fighting situation (AOR, 4.09; 95% CI, 2.52-6.66), and depression (AOR, 3.98; 95% CI, 2.21-7.16) were associated with PTSD. Participants with depression were almost 4 times more likely to have PTSD (AOR, 3.98; 95% CI, 2.21-7.16) than those without depression (Table 2).

**Table 1.** Description of 601 Respondents’ Clinical, Psychosocial, Substance, and Trauma-Related Factors in Amhara, Ethiopia, 2022

Variable	Frequency, No. (%)
Ever history of mental illness	
Yes	89 (14.8)
No	512 (85.2)
Ever treatment for mental illness	
Yes	72 (12.0)
No	17 (2.8)
Physical comorbidity	
Yes	118 (19.6)
No	483 (80.4)
Family history of mental illness	
Yes	153 (25.5)
No	448 (74.5)
Frequency of displacement	
One time	290 (48.3)
≥1 Time	263 (43.8)
Never	48 (8.0)
Ever use of substances	
Yes	271 (45.1)
No	330 (54.9)
Current use of substances	
Yes	253 (42.1)
No	348 (57.9)
Perceived stress level	
Low	11 (1.8)
Moderate	145 (24.1)
High	432 (71.9)
Depression	
Yes	177 (29.5)
No	424 (70.5)
Social support	
Low	177 (29.5)
Moderate	288 (47.9)
Strong	136 (22.6)
Stressful event	
No stressful event	84 (14.0)
1-2 Stressful events	181 (30.1)
≥3 Stressful events	336 (55.9)
Ever experienced abuse and neglect	
Yes	28 (4.7)
No	573 (95.3)
Destruction of personal property	
Yes	231 (38.4)
No	370 (61.6)
Lack of housing or shelter	
Yes	340 (56.6)
No	261 (43.4)
Lack of food or water	
Yes	404 (67.2)
No	197 (32.8)
Witnessing the murder of a family member/friend	
Yes	442 (73.5)
No	159 (26.5)
Ill health without medical care	
Yes	180 (30.0)
No	421 (70.0)
Forced isolation from other people	
Yes	202 (33.6)
No	399 (66.4)
Tortured or beaten	
Yes	174 (29.0)
No	427 (71.0)
Serious injury	
Yes	170 (28.3)
No	431 (71.7)
Witnessing the murder of a stranger	
Yes	83 (13.8)
No	518 (86.2)
Made to accept ideas against the will	
Yes	186 (30.9)
No	415 (69.1)
Forced separation from family	
Yes	288 (47.9)
No	313 (52.1)
Unnatural death of family or friends	
Yes	219 (36.4)
No	382 (63.6)
Imprisonment against the will	
Yes	41 (6.8)
No	560 (93.2)
Being in a warfighting situation	
Yes	403 (67.1)
No	198 (32.9)
Being abducted or kidnapped	
Yes	10 (1.7)
No	591 (98.3)
Rape or sexual abuse	
Yes	10 (1.7)
No	591 (98.3)

**Table 2.** Bivariable and Multivariable Logistic Regression Analysis of PTSD Among Conflict-Affected People in Amhara, Ethiopia, 2022

Explanatory variable	PTSD, No.		COR (95% CI)	AOR (95% CI)	*P* value^a^
	Yes	No			
Age range, y					
18-24	11	75	2.90 (1.40-6.00)	2.36 (0.78-7.15)	NA
25-34	55	184	1.42 (0.89-2.27)	0.94 (0.46-1.90)	NA
35-44	37	95	1.09 (0.64-1.84)	0.70 (0.34-1.41)	NA
≥45	43	101	1 [Reference]	1 [Reference]	NA
Sex					
Male	59	245	1 [Reference]	1 [Reference]	NA
Female	87	210	0.58 (0.39-0.84)	0.48 (0.27-0.86)	.01
Marital status					
Married	65	248	1 [Reference]	1 [Reference]	NA
Single	34	147	0.88 (0.55-1.40)	1.68 (0.84-3.34)	NA
Separated	12	15	0.28 (0.12-0.67)	0.49 (0.15-1.59)	NA
Divorced	17	34	0.46 (0.23-0.92)	0.91 (0.34-2.39)	NA
Widowed	18	11	0.14 (0.06-0.32)	0.43 (0.12-1.49)	NA
Occupational status					
Housewife	37	62	0.42 (0.22-0.79)	0.80 (0.31-2.03)	NA
Farmer	19	44	0.58 (0.28-1.20)	0.78 (0.29-2.12)	NA
Merchant	45	110	0.61 (0.34-1.11)	0.56 (0.25-1.28)	NA
Others	21	83	1.64 (0.86-3.12)	1.38 (0.55-3.44)	NA
Government employed	24	156	1 [Reference]	1 [Reference]	NA
Income, ETB					
<2995.35	98	232	0.51 (0.34-0.75)	0.76 (0.41-1.40)	NA
≥2995.35	48	223	1 [Reference]	1 [Reference]	NA
Experience of abuse					
Yes	10	18	0.56 (0.25-1.24)	1.70 (0.53-5.40)	NA
No	136	437	1 [Reference]	1 [Reference]	NA
Witnessing the murder of a family member/friend					
Yes	121	321	0.49 (0.30-0.79)	0.58 (0.32-1.03)	NA
No	25	134	1 [Reference]	1 [Reference]	NA
Experience of torture					
Yes	34	140	1.46 (1.05-2.25)	1.77 (1.02-3.08)	.04
No	112	315	1 [Reference]	1 [Reference]	NA
Family history of mental illness					
Yes	49	104	0.58 (0.39-0.88)	1.03 (0.61-1.75)	NA
No	97	351	1 [Reference]	1 [Reference]	NA
Witnessed murder of other people					
Yes	79	123	0.31 (0.21-0.46)	0.71 (0.39-1.27)	NA
No	67	332	1 [Reference]	1 [Reference]	NA
Ill without medical care					
Yes	66	114	2.46 (1.67-3.64)	0.60 (0.35-1.02)	NA
No	80	341	1 [Reference]	1 [Reference]	NA
Witnessed murder of stranger					
Yes	41	42	0.26 (0.16-0.42)	0.52 (0.26-1.04)	NA
No	105	413	1 [Reference]	1 [Reference]	NA
Experience of ideas against the will					
Yes	66	120	0.43 (0.29-0.63)	0.94 (0.55-1.60)	NA
No	80	335	1 [Reference]	1 [Reference]	NA
Unnatural death of family/friends					
Yes	75	144	0.43 (0.30-0.64)	0.63 (0.38-1.05)	NA
No	71	311	1 [Reference]	1 [Reference]	NA
Forced separation from family					
Yes	100	188	0.32 (0.21-0.48)	0.54 (0.30-1.99)	NA
No	46	267	1 [Reference]	1 [Reference]	NA
Being in a warfighting situation					
Yes	63	340	3.89 (2.63-5.75)	4.09 (2.52-6.66)	<.001
No	83	115	1 [Reference]	1 [Reference]	NA
Current substance use					
Yes	53	200	1.37 (0.93-2.02)	0.77 (0.46-1.29)	NA
No	93	255	1 [Reference]	1 [Reference]	NA
Depression					
Yes	27	169	2.60 (1.64-4.12)	3.98 (2.21-7.16)	<.001
No	119	286	1 [Reference]	1 [Reference]	NA

Abbreviations: AOR, adjusted odds ratio; COR, crude odds ratio; ETB, Ethiopian birr (US $52.27); NA, not applicable; PTSD, posttraumatic stress disorder.

^a^ The few P values reported reflect only the explanatory variables with significant results in the multivariable logistic regression analysis.

## Discussion

In the current study, we found that depression, the experience of torture or being beaten, being in a war situation, and being female was associated with PTSD. The presence of depression might be a factor in an increased risk of developing PTSD; people with depression could be more likely to have traumatic experiences than people without depression, potentially leading to an increase in the likelihood of having PTSD.^6^ Being female was associated with decreased risk of PTSD when compared with males. Males might be more likely to have higher exposure to potentially traumatic and war zone-related events such as the experience of unintentional injuries, assaults, witnessing death or injury, disaster or fire, and combat than females. This could be because males were more involved in the war and were the main focus of attention by the opponent’s force. Study limitations include a lack of national representativeness. Our findings suggest that interventions to manage these trauma-related events should be a priority to help conflict-affected communities.

## Notes

Supplement.
Data Sharing Statement
