# Supplementary material for: Posttraumatic Stress Disorder Among Residents of Conflict-Affected Towns
Source: JAMA Netw Open. 2023 Aug 16;6(8):e2329156. doi: 10.1001/jamanetworkopen.2023.29156 (PMC10433083; doi:10.1001/jamanetworkopen.2023.29156)
Supplement: Supplement. — Data Sharing Statement [file jamanetwopen-e2329156-s001.pdf]

Data Sharing Statement

Moges. Posttraumatic Stress Disorder Among Residents of Conflict-Affected Towns. *JAMA Netw Open*. Published August 16, 2023. doi:10.1001/jamanetworkopen.2023.29156

Data
Data available: No

Additional Information
Explanation for why data not available: The datasets used and analyzed during this study are available from the corresponding author upon reasonable request.
